# Structural Entropy: Monitoring Correlation-Based Networks Over Time With Application To Financial Markets

**DOI:** 10.1038/s41598-019-47210-8

**Published:** 2019-07-25

**Authors:** Assaf Almog, Erez Shmueli

**Affiliations:** Tel Aviv University, Department of Industrial Engineering, Tel Aviv, 69978 Israel

**Keywords:** Complex networks, Applied physics

## Abstract

The concept of “Structural Diversity” of a network refers to the level of dissimilarity between the various agents acting in the system, and it is typically interpreted as the number of connected components in the network. This key property of networks has been studied in multiple settings, including diffusion of ideas in social networks and functional diversity of regions in brain networks. Here, we propose a new measure, “Structural Entropy”, as a revised interpretation to “Structural Diversity”. The proposed measure relies on the finer-grained network communities (in contrast to the network’s connected components), and takes into consideration both the number of communities and their sizes, generating a single representative value. We then propose an approach for monitoring the structure of correlation-based networks over time, which relies on the newly suggested measure. Finally, we illustrate the usefulness of the new approach, by applying it to the particular case of emergent organization of financial markets. This provides us a way to explore their underlying structural changes, revealing a remarkably high linear correlation between the new measure and the volatility of the assets’ prices over time.

## Introduction

Following the 2008 financial crisis, there has been a soaring interest to broaden our understanding of financial markets’ behavior^1,2^. In particular, classical econometric models of rational agents and efficient markets were found to be very limited in explaining such extreme events, in part since they ignored complex interactions within the system^3^.

Bridging this gap, many recent studies have started to look at financial markets as complex systems with complex interactions between their various components^4–7^. One particularly interesting line of research, which was derived from this complex system perspective, is the representation of financial markets as correlation-based networks. Correlation-based networks are frequently used in fields such as neuroscience, biology and finance, as a way to infer a network structure from time series signals of the system’s components. In the case of financial markets, network nodes represent financial assets or instruments and network edges represent interactions between two assets, where such an interaction is typically measured by the correlation between the two assets’ price fluctuations over time.

The representation of financial markets as networks created a unique opportunity to strengthen our understanding of their behavior by applying standard network science tools. Indeed, various methods were suggested for inferring meaningful information about the markets by analyzing their network structure. A few examples include minimal spanning trees (MST)^4,8^, planar graphs^9,10^, asset trees^4,7,11^, and community detection for correlation matrices^12,13^. Recent studies in this field, took one step forward and explored structural properties of the financial networks in different time periods, including extreme events and economic crises. However, the majority of these studies share two main limitations: First, they commonly select a specific period of time, and construct the corresponding network out of it^4,7,8,11^. In some cases, several periods of time are chosen, such as in the case of before and after a financial crisis^14–16^. Nonetheless, this approach does not provide any information on the dynamic process that led from one static network to another. Second, the resulting network structure contains a substantial amount of information (this is especially true for large networks), and in many cases, extracting meaningful insights out of it is very challenging.

To address the two limitations mentioned above, we propose an approach for continuous monitoring of the structure of correlation-based networks, and demonstrate its application to the special case of financial markets.

We first introduce a new measure, “Structural Entropy”, as a revised interpretation to the “Structural Diversity” of a network. Structural diversity refers to the level of dissimilarity between the various agents acting in the network, and it is typically interpreted as the number of connected components in the network. This key property of networks has been studied in multiple settings, including diffusion of ideas in social networks^17,18^, and diversity of functional brain regions in Neuroscience^19,20^. In contrast, “Structural Entropy” is calculated based on the community structure of the network, which represents a finer grained division of the network into sub-units than in the case of simple connected components. Moreover, “Structural Entropy” takes into consideration both the number of communities and their sizes, encapsulating a richer and more meaningful representation of the network’s structure into a single value. The proposed measure was inspired by Shannon Index, which is commonly used in the ecological literature to provide some indication regarding the bio-diversity level of an ecosystem. Here, we generalize and adjust Shannon Index to quantify the structural diversity of complex networks.

We then suggest an approach for continuous monitoring of the structure of networks, which relies on the newly suggested structural entropy measure. Since structural entropy generates a single value that represents the network’s structure, it allows us to explore underlying structural changes in the network over time, in a relatively straightforward way. In this paper, we focus on correlation-based networks, where dynamic changes in the network structure are typically inherent. In particular, since the structure of such correlation-based networks is inferred from the activity of the system’s sub-units, monitoring the structural changes is highly important and can reveal underlying trends or phenomena in the system’s activity. For this aim exactly, we apply the structural entropy measure for continuous monitoring of correlation-based networks.

Finally, we illustrate the strength of the new approach, by applying it to the particular case of emergent organization of financial markets. In the context of financial markets, this organization translates into communities of stocks sharing the same price dynamics over time. More specifically, we construct assets-based correlation networks of two major financial markets, and monitor the structural entropy of these networks over time. Our analysis reveals a remarkably high linear correlation between the new measure and the volatility of the assets’ prices over time.

## Results

### Structural entropy

Real world complex networks are commonly organized in a modular way with communities of nodes that have dense connections internally and sparse connections externally^21^. These clusters represent the independent sub-units of the network, like families in social networks or brain regions in brain networks. Based on this community structure, we introduce “structural entropy” as a measure to quantify the level of structural diversity in a given network. In this framework, structural entropy refers to the level of heterogeneity of nodes in the network, with the premise that nodes that share functionality or attributes are more connected than others.

In practice, the measurement of structural entropy is composed of two main steps. The first step requires the identification of the network’s community structure, where each node is associated with a specific cluster (i.e. an optimal partition function). The second step includes the analysis of the partition function and the extraction of the diversity level as a single representing value.

We start by applying a community detection algorithm. This is mainly a generic step, nevertheless, different types of networks may require different approaches to optimally resolve their community structure. Over the years new various community detection and clustering techniques have been developed across different fields. Several specialized versions include algebraic topological data methods^22,23^ and methods based on a dedicated quality function like surprise maximization^24^. The selection of the community detection algorithm can be derived from the network properties such as size, density, directionality and more^25^. For the sake of simplicity, in this paper we focus on community detection algorithms that divide the network nodes into non-overlapping communities (i.e., each node is associated with exactly one community).

Let us now consider a network *G* with *N* nodes and let *A* be the chosen community detection algorithm. The partition of nodes into communities, as obtained by applying *A* on *G*, can be represented by an *N*-dimensional vector $\overrightarrow{\sigma }$, where the *i*-th component ${\sigma }_{i}$ denotes the community to which node *i* was assigned to. The values in $\overrightarrow{\sigma }$ range from 1 (community one) to *M* which is the total number of detected communities.

Given the partition $\overrightarrow{\sigma }$, we calculate the *M*-dimensional probability vector $\overrightarrow{P}$ which represents the proportional size of the clusters in the network

1 $$P\equiv [\frac{{c}_{1}}{N},\frac{{c}_{2}}{N},\,\mathrm{..},\,\frac{{c}_{M}}{N}]$$

where *c*_*i*_ is the size of community *i*. More specifically, the vector $\overrightarrow{P}$ represents the probability of randomly drawing a node from each community (note that ${\sum }_{i\mathrm{=1}}^{M}\,{P}_{i}=1$).

Finally, we apply Shannon entropy to the probability vector:

2 $$S\equiv H(P)\equiv -\,\sum _{i=1}^{M}{P}_{i}\,log({P}_{i})\mathrm{.}$$

and the resulting value is defined as the “structural entropy” of network *G*.

Structural entropy is calculated based on the number of communities and their sizes. The calculation does not take into account the internal structure of the communities. Consider a network of *N* nodes. The minimal value for the structural entropy of a network is 0, and this value is obtained when all nodes in the network are assigned to the same community (i.e., a single huge community). In contrast, the maximal value for the structural entropy of a network is obtained when each node is assigned to its own (different) community (i.e. *N* singleton communities). The value in this case depends on the number of nodes in the network *N*. If we fix the number of communities to a given number *C*, then the minimal value is obtained in the case of *C* − 1 singleton communities and a single large community with *N* − *C* + 1 nodes. Similarly, the maximal value is obtained in the case of *C* communities, each having *N*/*C* nodes.

**Figure 1:**
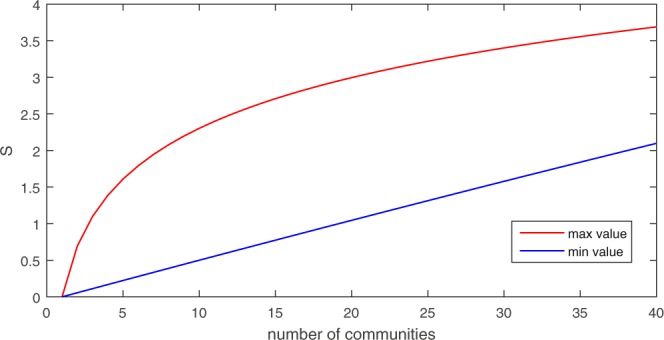
Minimal (blue) and maximal (red) structural entropy values for networks with a thousand nodes and a varying number of communities.

To illustrate the possible range of values of Structural Entropy, we plot in Fig. 1 the maximal and minimal structural entropy values for networks that consist of 1000 nodes and a varying number of communities. The red line represents the maximal value and the blue line represents the minimal value of the structural entropy as a function of the number of communities. Clearly, in realistic network configurations, the actual structural entropy value will be somewhere in between these two lines.

The structural entropy measure encapsulates considerable amount of information regarding the community structure of the network in a single value, and can serve as a valuable indicator in different domains. In particular, the proposed measure is analogous to (and inspired by) the Shannon Index, which is commonly used in the ecological literature, as an indicator of the diversity level in an ecosystem. Here, we essentially adjust the Shannon Index to the setting of complex networks to measure structural diversity. In other words, we measure the diversity level as emerged from the structure of the network, i.e. size and number of communities, as obtained by a community detection algorithm. The measure quantifies the diversification in the network in terms of connectivity, i.e. the level of node fragmentation to different groups (communities) in the network. While Shannon Index gives a general indication to the current state of a system, the index itself is not explicitly informative. In contrast to the common use of Shannon Index in ecology, we want to analyze the dynamics of our proposed measure as it evolves over time.

### Continuous monitoring of correlation-based networks

Monitoring the dynamics of structural entropy over time can reveal significant information on underlying processes in the system. In this section, we propose a general approach for the continuous monitoring of structural entropy for the specific case of correlation based networks.

A correlation-based network represents a system’s network structure as derived by time series activity of the agents in the system. This approach is commonly used in fields such as Neuroscience, Finance, and Biology, where inferring information from empirical observation is vital. The aim is the extraction of meaningful information form multiple time series data such as: neural activity, stock prices, metabolic profiles. These multiple time series allow us to infer and identify emergent network organization of the system based on the activity of each component. Specifically, because the structure of correlation-based networks is inferred from the units activity, monitoring the structural changes is very important and can reveal underlying trends or phenomenons in the system.

For this aim, we describe next, a general framework for continuous monitoring of structural entropy in correlation-based networks. In particular, we specify the process to extract time dependent structural entropy from the empirical data, provided as multiple time series.

Let us consider a system with *N* units. The single time series:

3 $${S}_{i}\equiv [{s}_{i}(1),\,{s}_{i}(2),\,{s}_{i}(3),\,\ldots ,\,{s}_{i}(T)]$$

represents the temporally ordered activity of the *i*-th unit of the system over *T* consecutive time steps. The set of time series for all *N* units, i.e., $\{{S}_{1},{S}_{2},\,\ldots ,\,{S}_{N}\}$, describes the synchronous activity of all units in the system.

Similarly to other studies in this field, we exploit the information encoded in the *N* × *N* cross-correlation matrix. The cross-correlation matrix *C* measures the mutual dependencies among the *N* time series on a scale between 1 and −1. The *ij*-th entry of *C* denoted by *C*_*ij*_ is defined as the Pearson correlation coefficient between the two time series *S*_*i*_ and *S*_*j*_:

4 $${C}_{ij}\equiv CORR[{S}_{i},{S}_{j}]\equiv \frac{COV[{S}_{i}][{S}_{j}]}{\sqrt{VAR[{S}_{i}]VAR[{S}_{j}]}}$$

where:

- $COV[{S}_{i}][{S}_{j}]\equiv \overline{{S}_{i}{S}_{j}}-{\bar{S}}_{i}{\bar{S}}_{j}$ is the co-variance between *S*_*i*_ and *S*_*j*_.
- $VAR[{S}_{i}]\equiv \overline{{S}_{i}^{2}}-\overline{{S}_{i}^{2}}=COV[{S}_{i}][{S}_{i}]$ is the variance of *S*_*i*_.
- $$\overline{{S}_{i}}\equiv \frac{1}{T}{\sum }_{t\mathrm{=1}}^{T}{s}_{i}(t)$$
- $$\overline{{S}_{i}^{2}}\equiv \frac{1}{T}{\sum }_{t\mathrm{=1}}^{T}{s}_{i}^{2}(t)$$
- $$\overline{{S}_{i}{S}_{j}}\equiv \frac{1}{T}{\sum }_{t\mathrm{=1}}^{T}{s}_{i}(t){s}_{j}(t)$$

Here, we suggest to measure the Pearson correlation for sub-periods of the time series data, using a’sliding window’ technique. We divide the original time series with length *T* to $T-\tau$ sub periods, where $\tau$ is the length of the sub-periods (the number of time steps within a window).

Then, we calculate the correlation matrix for time step t, denoted by *C*^*t*^, as:

5 $${C}_{ij}^{t}\equiv CORR[{S}_{i}(t-\tau ,t),{S}_{j}(t-\tau ,t)]$$

Once the correlation matrix has been constructed (for each sub-period), the next step is transforming it into an adjacency matrix. The adjacency matrix is the representation of the network edges, and can be extracted from the correlation matrix in different ways. The most common approach is to use some threshold criteria, which determines which values of the correlation matrix will be transformed into edges in the network (and which values will not). However, there are other more refined ways to extract the adjacency matrix from the correlation matrix is used random matrix theory^12,13^, and choosing which method use depends on the properties of the specific data-set in hand.

Once the adjacency matrix (of the specific sub period) is resolved, we measure the structural entropy as described in the previous section. Note that Structural Entropy is calculated in each time step for the corresponding sub-period, resulting in a new time series of Structural Entropy values.

**Figure 2:**
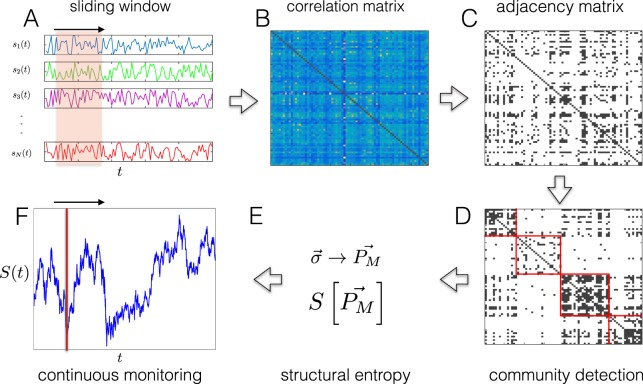
Illustration of the main steps in the procedure of monitoring structural entropy of correlation-based networks. (**A**) raw time series, using sliding window approach we analyze continuously sub-periods from the entire data. (**B**) generating Pearson correlation matrix (or different association matrix) (**C**) transforming the correlation matrix to an adjacency matrix (**D**) resolving community structure of the network (**E**) calculating the structural entropy for each specific sub-period (**F**) continuous monitoring of the structural entropy and analysis.

Figure 2 depicts the main steps in the procedure of monitoring structural entropy of correlation-based networks. In sub-figure A we observe the raw data and the use of a sliding window technique. Next, in sub-figure B, we calculate the Pearson correlation matrix from the raw data. In sub-figure C, we see the transformation of the correlation matrix into an adjacency matrix using a threshold process. These three stages represent the main steps discussed in this section. The next two steps show the calculation of structural entropy. In sub-figure d, we see the outcome of a community detection algorithm running over the adjacency matrix, where each block in the matrix represents a community with high density of links. Next, in sub-figure E, we see the calculation of structural entropy as described in section 0. Lastly, in sub-figure F, we construct a new time series for the structural entropy measure, where each time step represents the diversity level of the system in the corresponding sub-period.

In the next section, we apply this process to the case of financial data in order to resolve and monitor diversity level in emergent organization of financial markets.

### Monitoring structural entropy in financial markets

For our analysis we use daily closing prices from the FTSE100 and NIKKEI225 indices, for a 10-year period ranging from 24/10/2001 to 18/10/2011. For each index, we retained in our data set only stocks that were traded continuously throughout the entire selected period. This results in 78 stocks for FTSE100 and 193 stocks for NIKKEI225. For each stock *s*_*i*_ in the data set, we constructed a time series which is composed of its daily log-returns (i.e. the the log of its daily increment), as commonly done in the financial literature:

6 $${s}_{i}(t)\equiv \,\mathrm{ln}(\frac{{p}_{i}(t)}{{p}_{i}(t-\mathrm{1)}})$$

where ${p}_{i}(t)$ is the daily closing price of stock *i* at time *t*.

Then, as explained in the previous section, we use a sliding window approach, where each sub-period was analyzed by constructing the corresponding correlation-based network. The selection of an appropriate time period length, from which the correlation matrix is computed, is a well-recognized problem in the domain of correlation based networks. Clearly, there is a trade-off between long and short periods A long period reduces fluctuations and noise but suffers from non-stationarity. In contrast, a short time period results in very singular correlation matrices with strong fluctuations.

In this paper we picked the ratio $\tau \approx 2N$, which is typically used in similar studies to balance the aforementioned trade-off. Thus, for the FTSE100 index we used a window (sub-period) of 200 trading days (approximately one calendar year), and for the NIKKEI225 we used a window (sub-period) of 400 trading days (approximately two calendar years). The difference in the two window sizes is a result of the different number of stocks monitored in each market (different *N*). However, we should stress that our findings are robust and consistent when using other period lengths $\tau$ which grater than the size of the system (i.e., $\tau  > N$) as we demonstrate later in this section.

In order to resolve the community structure, we used a recent method^12,13,26^, which is specifically shaped to deal with correlation matrices, and is based on random matrix theory (for more information see the Methods section). The method infers the communities directly from the correlation matrix using a random null model, by filtering out the system noise and global trends. This particular capability allows us to explore the system with very short time windows $N < \tau  < 3N$, and it is the main reason why our findings are invariant to to the size of the window. More specifically, using random matrix theory the method filters the relevant noise based on a specific null model.

In general, other approaches, such as clustering (e.g., DBSCAN^27^), can be applied on the correlation matrix to extract the communities. However, in our setting, the correlation matrix is very dense, and applying such methods will typically result in a single community. To cope with this problem, it is possible to use some threshold criteria to determine which values of the correlation matrix will be transformed into edges in the network and which values will not. However, the threshold approach presents several major limitations as we further describe in the appendix.

In recent years, random matrix theory has become a popular tool for investigating the dynamics of financial markets using cross-correlations of empirical return time series^28^. For example, a recent work by Pharasi *et al*.^29^, used a power map to filter the noise from extremely short time frames and identify markets states. The researchers have isolated different independent markets states and analyzed the transition probability from one state to the other. Here, we take a different approach based on a continuous sliding window rather than independent “snapshots” of the system. In the sliding window approach, two consecutive time sub-periods share almost the exact same information by construction. Since financial systems are known to have a very short memory and to contain a significant amount of noise, this is a very accepted procedure.

We should also make a clear distinction with respect to different information theory measures such as mutual information^30^ and transfer entropy^31,32^, which were used to quantify information transfer between two random processes. These measures are used frequently in the studies of temporal networks, when the time-varying changes are of a particular relevance to spreading processes, like the spread of information or disease. It is important to stress that our approach is invariant to the specific composition and changes in the clusters. More specifically, structural entropy does not aim at quantifying the information transferred between two consecutive sub-periods. It rather adopts a more “grand canonical” framework, and does not focus on the specific variations in the links. In other words, structural entropy is calculated based on the number and size of clusters only, regardless of which node (stock in this case) belongs to which cluster.

Clearly, mutual information can also be used in our case as a nonlinear similarity measure instead of Pearson correlation^33^. However, for the type of data considered in this section, i.e. time series of stocks, the typical measure used in the literature is Pearson correlation. This is due in part to the following reasons: (1) time series of stocks have extremely short memory and the main assumption is that each time step is completely independent from the others, and (2) the dynamics itself is highly correlated on one hand and noisy on the other hand, where the main challenge is to filter the high level of correlation and “purifying” significant information.

In the context of correlation based networks, another entropy measure that should be discussed is spectral entropy, which aims at measuring the spread of functionalists in a correlation matrix. In particular, spectral entropy measures the entropy of the power spectral density (the matrix eigenvalues). This measure appears to share some resemblance with the proposed structural entropy measure (in the specific case of correlation based network), since the matrix is decomposed and then filtered based on the eigenvalues. However, our method is inherently different. First, the random matrix theory method we use, filters the random noise and discards the majority of the eigenvalues (as shown in Fig. 11). Thus, the majority of data used in the calculation of spectral entropy is discarded as noise (and global mode). Second and more important, once the “noisy” eigenvalues are identified, a “filtered network” is re-constructed. This means that the detected community structure is a result of a super-position of all the non-random eigenvalues and cannot be attributed to the number and magnitudes of the eigenvalues. For instance we cannot easily connect the number of non-random eigenvalues to the number of communities. In the appendix, we make a more thorough comparison between the two measures and present the results of this comparison.

We start our analysis by exploring the complete network structure in the two sub-periods with the maximal and minimal structural entropy. This step demonstrates the different’structural states’ of a market as identified by the new measure.

**Figure 3:**
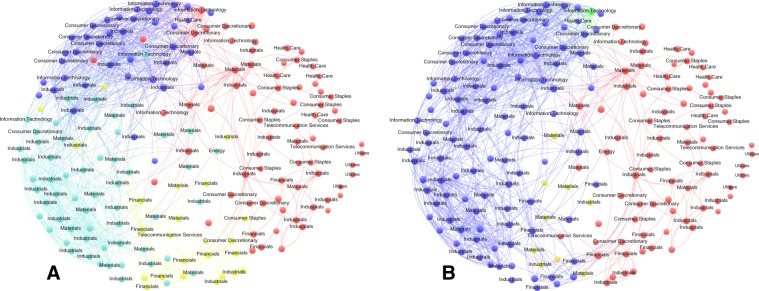
The NIKKEI market structure for the sub-periods with the highest entropy $S=0.2628$ (right) and the lowest entropy $S=0.1327$ (left). The values are normalized with the maximum entropy value when the system is composed of $N=193$ communities. The different colors represent the different communities detected by each run, where the labels of the node represent the industry sector the stock belongs to according to the GICS classification.

Figure 3 depicts the NIKKEI community structure of the two sub-periods with the maximal (A) and minimal (B) structural entropy throughout the 10 year period. Each stock is represented as by a dot, the 4000 highest correlations in the matrix are shown as edges, and different communities are colored differently. As shown in the figure, each of the detected communities contains a highly clustered core, i.e. the core has higher values of correlation with respect to the rest of the values in the community. Surprisingly, despite the major difference in the structural entropy of the two sub-periods, both sub periods contain exactly 4 communities. This is because the sizes and the profiles of the communities in the two sub-periods are very different.

To better understand the result above, we label each of the stocks according to its industry sector using the Global Industry Classification Standard (GICS). As can be seen in the figure, the blue and light blue communities in Subfigure A, which mainly contain the sectors: industrials, Consumer Discretionary, and Materials, are converged into one community in Subfigure B, which also observe some additional stocks. The Yellow community in Subfigure is reduced to contain a small number of stocks in Subfigure B, and the green cluster in Subfigure B is composed out of a single stock. This result provides a strong support for our proposed measure, where it demonstrates how the information about the number of communities is not sufficient and more information is needed to describe the structural diversity of the system.

Next, we explore the presence of the financial crisis in the data with conventional measurements. Volatility is a statistical measure of the dispersion of returns for a given market index (e.g. FTSE, NIKKEI). This measure refers to the level of uncertainty or risk associated with the size of changes in the market. A high volatility level corresponds to a high range of fluctuations in the prices of the stocks. This means that the price of an asset can change dramatically over a short time period in either direction. A lower volatility means that the asset value does not fluctuate dramatically, and tends to be more steady. Here, we define the volatility as the variance of all the log-returns in a given sub-period:

7 $$VAR[{r}_{i}(t)]\equiv \overline{{[{r}_{i}(t)]}^{2}}-{\overline{[{r}_{i}(t)]}}^{2}\mathrm{.}$$

**Figure 4:**
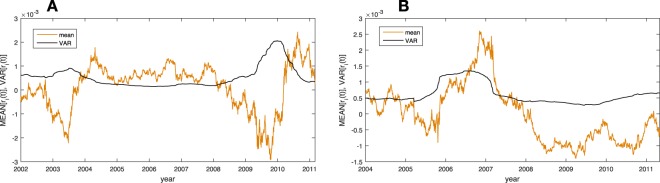
The average log-return (orange) and volatility (black) for each sub-period for FTSE100 (**A**) and NIKKEI225 (**B**) for approximately 10 years from 2001Q4 to 2011Q4. We can clearly see the relationship between high volatility and fluctuations in log-returns.

In Fig. 4, we plot the measured volatility (black) and the mean log-return (orange) over the sub-periods for the FTSE100 and NIKKEI225 index (the X axis represents the date of the last day in the sub-period). The evidence of the crisis is very clear, where the shift in the time of the crisis are a result of the window sizes used (200 and 400 days) and the difference in the markets. We can also observe a clear connection between high volatility to drastic fluctuations in log returns.

After confirming the presence of the extreme event in our data, we turn to exploring the relation between the market’s volatility and the structural diversity of the market. We anticipate that the different price dynamics in each sub-period would lead to a different community structure and in turn to a different structural entropy value.

**Figure 5:**
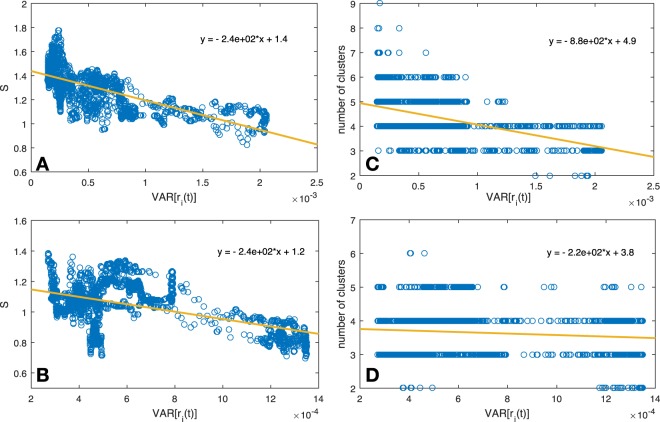
We plot the relation between volatility (X axis) and structural entropy (Y axis) for FTSE100 (**A**) and NIKKEI225 (**B**). For reference we also plotted the relation between volatility (X axis) and the number of detected communities (Y axis) for FTSE100 (**C**) and NIKKEI225 (**D**).

In Fig. 5, we present the relation between volatility (X axis) and structural entropy (Y axis) for FTSE100 (top left panel) and NIKKEI225 (bottom left panel). We find a very strong anti-correlation between the two measures, which is present in the two markets (FTSE100 corr = −0.701, NIKKEI225 corr = −0.426). Indeed, we expected such negative relation, which reinforces the known behavior of markets to cluster (reduce structural diversity) in times of strong volatility. Surprisingly however, the remarkable result lies within the nicely fitted linear relation. More specifically, we can see that the sub-periods with the highest volatility (crisis times) do not appear as outliers, but rather fit nicely on the line. For reference we also plotted the relation between volatility (X axis) and the number of detected communities (Y axis) for FTSE100 (top right panel) and NIKKEI225 (bottom right panel). Unlike the relation described above, we did not find a clear relation between the number of communities and volatility.

**Figure 6:**
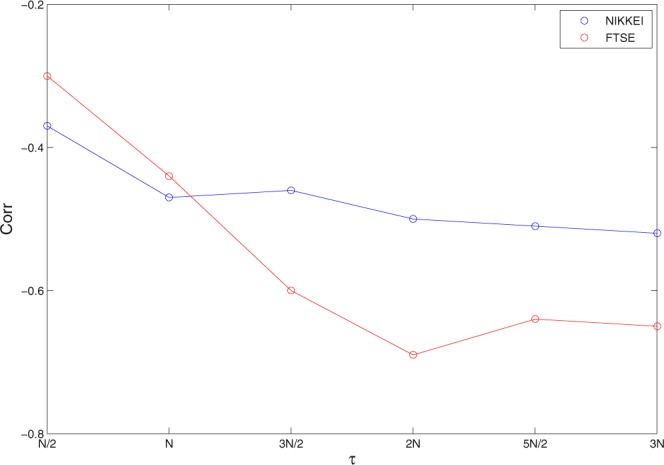
The correlation between structural entropy and volatility for different (sliding) window sizes in both markets. As can be seen, high anti-correlation values appear at $\tau  > N$ and the fluctuations seem to stabilize at $\tau =2N$.

We further analyze the relation between structural entropy and volatility in both markets for different window sizes. Figure 6 presents the correlation values between structural entropy and volatility *Corr*[*Var*, *SE*] for different lengths of the sliding window. As can be seen from the figure, the high anti-correlation relation becomes evident already at $\tau  > N$. However, these values present some fluctuation which seem to stabilize around $\tau =2N$.

**Figure 7:**
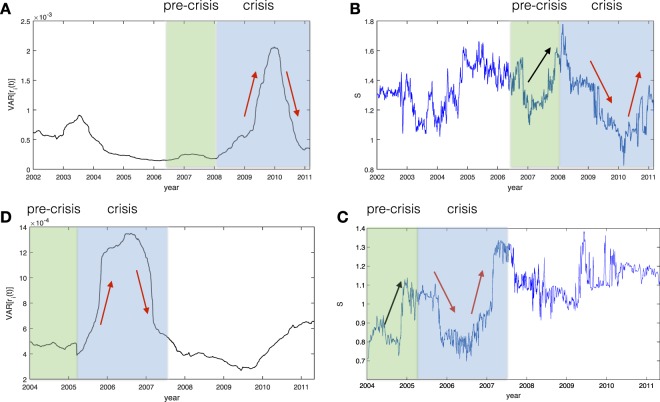
Measured volatility (**A**) and structural entropy (**B**) for FTSE100. Measured volatility (**C**) and structural entropy (**D**) for NIKKEI225).

The previous result showed the strong negative relation between the structural entropy and the volatility in the system. However, it is more interesting (and beneficial) to analyze the structural entropy as it evolve over time, i.e. the dynamics of structural entropy. In Fig. 7, we plot the values of the structural entropy for 10 years (right panel) and the corresponding volatility (left panel). While the structural entropy measure is associated with some noise, we can still observe clear trends and shifts in the diversity of the system. We highlight the crisis period (in light blue) and the pre-crisis period (in light green) as observed by the volatility in the data. In the crisis period, we indeed see a negative relation between the two measures as highlighted by the red arrows. Interestingly, in the pre-crisis period we can see different behaviors: while structural entropy presents a big shift (in the same order of magnitude as in the crisis) volatility remain roughly constant. This may suggest that structural entropy is able to detect a significant change in the community structure ahead of time, while volatility is invariant to those changes.

**Figure 8:**
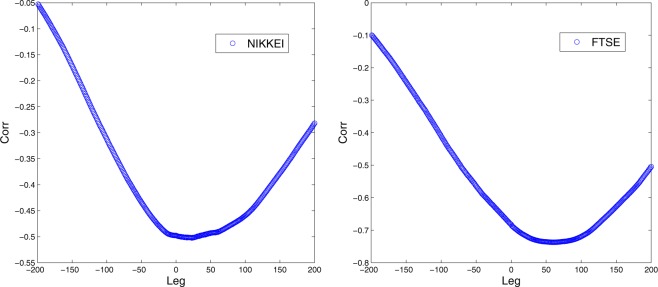
The correlogram between structural entropy and volatility for the NIKKEI market (left) and the FTSE market (right).

To further analyze this relation we calculate the correlogram of structural entropy and volatility (see Fig. 8). This analysis reveals an interesting relation between the two measures, where in both examined datasets, the correlation reaches the highest value with a positive leg between structural entropy and volatility. The effect is significantly more evident in the FTSE market where the delay around 90 days. This result might suggest some level of predictive ability of the proposed measure, and it opens the door for future research with respect to the proposed measure.

## Conclusion

In this study, we proposed a robust approach to quantify and monitor the structural diversity of correlation-based networks. At the heart of the approach lies the newly introduced “Structural Entropy” measure, which utilizes the finer-grained network communities (in contrast to the network’s connected components), and takes into consideration both the number of communities and their sizes. The proposed approach can serve as a powerful analysis tool in different settings, with the ability to combine various structural properties of a network into one representing value, and allowing to monitor these values over time.

We further demonstrated how the proposed approach can be applied to the particular case of monitoring structural diversity in emergent organization of financial markets. We showed that structural entropy can be used to differentiate structural states of the financial markets, and even found a strong linear relation between structural entropy and volatility of the system. These observations were consistent across different markets and periods.

Finally, we observed that our proposed measure can detect trends that could not otherwise be detected by volatility, indicating that it might be useful as an early warning signal to future major changes in financial markets and perhaps even in other settings. Clearly, testing this idea on additional markets, and building a predictive model based on these signals are very interesting directions for future research.

Our approach takes a simplified perspective of structural diversity which analyzes a network based on linear pairwise correlation. We find the simplicity of the approach to be a major advantage, since it can be used in a relatively interpretable manner by a wide variety of disciplines such as economy, neuro-science, biology, etc. (and is not restricted to physics or mathematics).

While our approach is suitable in cases where the community structure of the network changes constantly over time, it is less suitable in cases where the network’s structure is more stable, such as some types of social networks in which the community structure is based on certain social factors^34^, or brain networks in which the community structure is strictly based on anatomy^35^. In both of these cases, the communities support the dynamic processes over the network rather than changing their number and size over time.

Moreover, the proposed structural entropy measure takes into account only the number of communities and their sizes, and disregards the internal structure of these communities and the connections between them. While it provides a relatively simple and interpretable quantification of the network structure, in some cases these internal structures and connections may hold highly nontrivial and important information. In this regard, it is worth noting other measures that were suggested in the literature and utilize such information, such as the one suggested by Andjelković *et al*. for the analysis of time-series graphs representing the traffic fluctuations on networks^36^ and the one by Garcia-Martinez *et al*. for extracting insightful information from brain activity signals^37^.

## Methods

### Community detection

**Figure 9:**
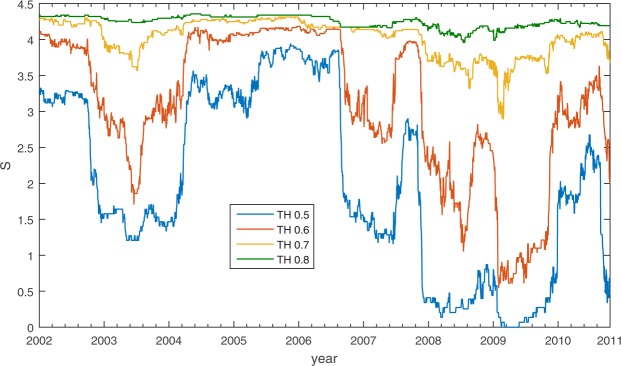
Measured structural entropy for the FTSE index using the popular threshold procedure. We can see that the results are highly sensitive to the value of the threshold.

For the community detection step, we adopt a new method^12,13^, which is specifically shaped to deal with correlation matrices, based on random matrix theory (for more information see the Methods section). By using the method we overcome two main limitations of more traditional clustering approaches^12^. First, the method does not requires introduction of any arbitrary threshold criteria, which can change the outcome of resolved structure dramatically as shown in Fig. 9. Secondly, the method filters statistical noise, thus, enables us to use shorter periods of time for our analysis.

Now, we use the redefined modularity measure for correlation matrices^12^. This redefinition avoids the use of a network representation and uses an appropriate null model that can be applied directly to correlation matrices. The method defines the modularity as

8 $$Q(\overrightarrow{{\boldsymbol{\sigma }}})=\frac{1}{{C}_{norm}}\sum _{i,j}\,[{C}_{ij}-{\langle {C}_{ij}\rangle }_{null}]\delta ({\sigma }_{i},{\sigma }_{j})$$

where *C*_*ij*_ is the correlation matrix and ${\langle {C}_{ij}\rangle }_{null}$ is a random null model that needs to identify the random properties of empirical correlation matrices.

In this approach, the empirical correlation matrix is first decomposed and then reconstructed using only the eigenvalues (and eigenvectors) that are not reproduced by the random null model. Thus in this context, we are interested in the correlation matrix spectrum which the random model (multiple time series) generates. Once compared with the observed spectrum of the empirical correlation matrix, the model will identify the non-random eigenvalues (by elimination). The non-random eigenvalues will later be used to generate the new filtered matrix. The null model serves as the “random benchmark” in this new definition of modularity.

Here, we use a null model to serve as the random benchmark for the empirical data. The null model describes the most simple case of a random system, where we have *N* independent, random time series for *T* time steps (the observed period). In this specific case, the resulting correlation matrix would be an *N* × *N* Wishart matrix, whose statistical properties are well-known^38,39^. In the limits where $N,T\to \infty$ and $T/N\ge 1$ the eigenvalues of the Wishart matrix are distributed according to a Marchenko-Pastur distribution

9 $$P(\lambda )=\frac{T}{N}\frac{\sqrt{({\lambda }_{+}-\lambda )(\lambda -{\lambda }_{-})}}{2\pi \lambda }\,\,if\,\,{\lambda }_{-}\le \lambda \le {\lambda }_{+}$$

and $P(\lambda )=0$ otherwise. The boundaries *λ*_+_ and *λ*_−_ are dependent on the data size and given by

10 $${\lambda }_{\pm }={[1\pm \sqrt{\frac{N}{T}}]}^{2}\mathrm{.}$$

This analytic curve represents the boundaries of the bulk eigenvalues, which predominantly represent noise, and so have little meaning assigned to them.

**Figure 10:**
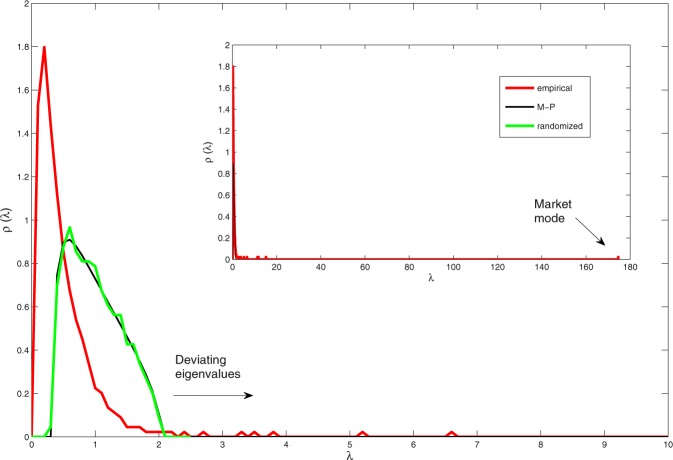
The eigenvalue density distribution of the empirical correlation matrix of the *S* & *P*500. Generated by daily closing prices from 2001Q4 to 2011Q3 for N = 445 stocks of the S&P500 index. In red is the empirical density distribution, in black is the theoretical Marchenko Pastur (M-P) prediction for a random correlation matrix (Eq. (9)), and in green is the eigenvalue density obtained by the randomized data. The inset is the fully zoomed-out version of the plot, showing the maximal eigenvalue (the ‘market mode’), as well as several deviating eigenvalues from the predicted curve.

In Fig. 10 we plot the eigenvalue density distribution of the empirical correlation matrix of the *S & P500*. In red is the empirical density distribution, in black is the theoretical Marchenko Pastur (M-P) prediction for a random correlation matrix (Eq. (9)), and in green is the eigenvalue density obtained by the randomized data. It is clear that the randomized curve is almost identical to the theoretical Marchenko Pastur distribution, confirming the agreement with random matrix theory for uncorrelated data. In the inset we can observe the maximal eigenvalue (the ‘market mode’), as well as several deviating eigenvalues from the predicted curve. The ‘market mode’ effect is caused by the fact that stocks typically move up or down together, this results in the presence of a very large eigenvalue ${\lambda }_{m}$, orders of magnitude greater than the rest. We can also see that a significant amount of the eigenvalues is laying in the ‘sub random’ range^12^, where $\lambda  < {\lambda }_{-}$. This phenomenon is associated with the presence of the global mode, where the random bulk is shifted to the right due to a very large eigenvalue and is discussed in^39^. Here we take the same rigorous approach and associate the ‘sub random’ range as noise, while the other deviating eigenvalues outside of the “random bulk“, have structural implications and relate to groups of correlated stocks^39^.

**Figure 11:**
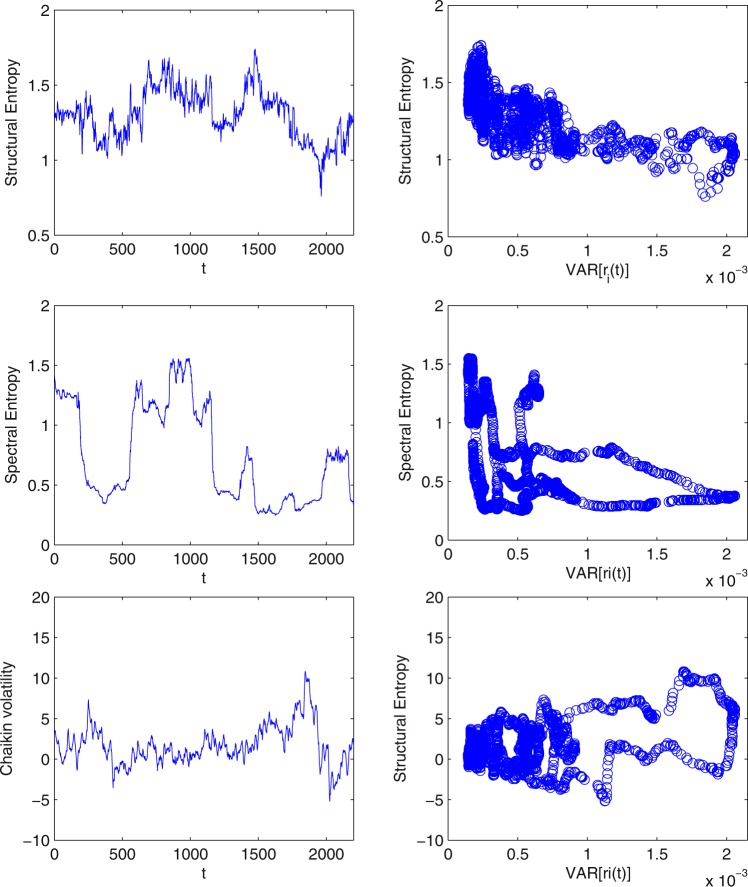
A comparison of Structural Entropy, Spectral Entropy and Chaikin volatility. In the left panels we plot the dynamics of all three measures over time. In the right panels we plot the relation between each measure and volatility for each sub-period.

As a result, any empirical correlation matrix *C* can be identified as the sum of three matrices:

11 $$C={C}^{(r)}+{C}^{(g)}+{C}^{(m)},$$

where *C*^(*r*)^ is the random part aggregated from the eigenvalues in the random spectrum (${\lambda }_{-}\le \lambda \le {\lambda }_{+}$), i.e.

12 $${C}^{(r)}\equiv \sum _{i:{\lambda }_{-}\le {\lambda }_{i}\le {\lambda }_{+}}{\lambda }_{i}|{v}_{i}\rangle \langle {v}_{i}|,$$

and

13 $${C}^{(m)}\equiv {\lambda }_{m}|{v}_{m}\rangle \langle {v}_{m}|$$

represents the market mode, and

14 $${C}^{(g)}\equiv \sum _{i:{\lambda }_{+} < {\lambda }_{i} < {\lambda }_{m}}{\lambda }_{i}|{v}_{i}\rangle \langle {v}_{i}|$$

represents the remaining correlated groups. These sub-groups of correlated stocks comprise the mesoscopic structure of the market. They are also referred to as “group modes” in the literature^4,39^.

The maximal eigenvalue represents a common factor influencing all the stocks in a given market, from a structural perspective, the market mode eigenvalue signifies the presence of one single super-community, containing all the stocks in the market. Thus, the other eigenvalues (not including the market mode), which deviate from the bulk, ${\lambda }_{+} < {\lambda }_{i} < {\lambda }_{m}$ are the ones corresponding to mesoscopic clusters, i.e. groups of stocks with similar dynamics. Now, returning to the modularity, we define the filtered empirical correlation matrix as ${C}_{ij}^{(g)}$ once both the global mode *C*^(*m*)^ and the random bulk *C*^(*r*)^ have been filtered.

Once we input this result into the modularity equation

$$Q(\overrightarrow{{\boldsymbol{\sigma }}})=\frac{1}{{C}_{norm}}\sum _{i,j}\,[{C}_{ij}-{\langle {C}_{ij}\rangle }_{null}]\delta ({\sigma }_{i},{\sigma }_{j})=\frac{1}{{C}_{norm}}\sum _{i,j}({C}_{ij}-{C}_{ij}^{(r)}-{C}_{ij}^{(m)})\delta ({\sigma }_{i},{\sigma }_{j})$$

we see that this leads to

15 $$Q(\overrightarrow{{\boldsymbol{\sigma }}})=\frac{1}{{C}_{norm}}\sum _{i,j}\,{C}_{ij}^{(g)}\delta ({\sigma }_{i},{\sigma }_{j}\mathrm{).}$$

In other words, to clearly differentiate between the mesoscopic groups, one must subtract out the main drift of the system and the random correlation, using the random null model. The filtered matrix ${C}_{ij}^{(g)}$ constituted from the “non-random” eigenvalues ${\lambda }_{+} < {\lambda }_{i} < {\lambda }_{m}$ and their corresponding eigenvectors ${v}_{i}$. The method modified three modern community detection algorithms, customizing where necessary to be effective with correlation matrices^12^.

Lastly, to broaden our analysis we compare the proposed structural entropy to the spectral entropy and Chaikin volatility. The first method aims at measuring the spread of functionality in a correlation matrix and shares some resemblances by using eigenvalues analysis. The latter, is a volatility indicator which calculates the Exponential Moving Average of the difference between the current interval’s high and low prices and its value a number of periods ago. We calculate each of the measures for the exact same sub-periods and using the same sliding window technique. In Fig. 11, we present a comparison between the three measures. In the panels on the left, we plot the dynamics of all three measures over time. In the panels on the right, we plot the relation between each measure and volatility for each sub-period. We can see that the spectral entropy has a very different dynamics than the structural entropy, and that its relation to volatility is very noisy. This is quite expected as spectral entropy does not filter noise and analyzes the whole spectrum of the correlation matrix. As for the Chaikin volatility measure, while it does not present a clear relation to the volatility measure, we can still observe the main event (volatile period). Nevertheless, structural entropy presents this volatile period long before it can be observed by Chaikin volatility^33^.
